# Immediate-Release Formulations Produced via Twin-Screw Melt Granulation: Systematic Evaluation of the Addition of Disintegrants

**DOI:** 10.1208/s12249-021-02056-0

**Published:** 2021-06-16

**Authors:** Kristina E. Steffens, Karl G. Wagner

**Affiliations:** Department of Pharmaceutics, University of Bonn, Gerhard-Domagk-Str.3, 53121 Bonn, Germany

**Keywords:** twin-screw melt granulation, immediate release, tablets, disintegration, dissolution

## Abstract

The current study evaluated the effect of location and amount of various superdisintegrants on the properties of tablets made by twin-screw melt granulation (TSMG). Sodium-croscarmellose (CCS), crospovidone (CPV), and sodium starch glycolate (SSG) were used in various proportions intra- and extra-granular. Tabletability, compactibility, compressibility as well as friability, disintegration, and dissolution performance were assessed. The extra-granular addition resulted in the fasted disintegration and dissolution. CPV performed superior to CCS and SSG. Even if the solid fraction (SF) of the granules was lower for CPV, only a minor decrease in tabletability was observed, due to the high plastic deformation of the melt granules. The intra-granular addition of CPV resulted in a more prolonged dissolution profile, which could be correlated to a loss in porosity during tableting. The 100% intra-granular addition of the CPV resulted in a distinct decrease of the disintegration efficiency, whereas the performance of SSG was unaffected by the granulation process. CCS was not suitable to be used for the production of an immediate-release formulation, when added in total proportion into the granulation phase, but its efficiency was less impaired compared to CPV. Shortest disintegration (78 s) and dissolution (Q_80_: 4.2 min) was achieved with CPV extra-granular. Using CPV and CCS intra-granular resulted in increased disintegration time and Q_80_. However, at a higher level of appx. 500 s and appx. 15 min, only SSG showed a process and location independent disintegration and dissolution performance.

**Supplementary Information:**

The online version contains supplementary material available at 10.1208/s12249-021-02056-0.

## INTRODUCTION

Melt granulation is an alternative granulation method to formulate powders that are not suitable for direct tableting because of their poor flowability or compactibility. Since melt granulation does not require the addition of solvents, the process is also interesting for water-sensitive compounds (1). The granules are formed by using a meltable binder (like waxes or polymers) in a heatable process equipment. Different techniques are approved, which include fluid-bed granulators, high shear mixers, and twin-screw extruders (2–11). With growing interest in continuous processing methods, twin-screw melt granulation (TSMG) became an attractive technique to granulate active pharmaceutical ingredients (APIs) especially, as a process time limiting drying step is avoided (7). Furthermore, in TSMG, only a very low excipient content is needed, resulting in high drug load final products (8). However, compared to wet granules, no pore formation by the removal of water can be observed. Therefore, melt granules show low porosities, especially when obtained from high shear granulation processes, like TSMG. The potential of this method to achieve an improved tabletability was often reported in literature (8, 9, 12, 13). This improved tabletability is mainly derived from the obtained closer binder network in the tablets, which simultaneously causes a low porosity of the produced compacts (14). Due to the strong relationship between the relative density and disintegration, the disintegration of these compacts might be limited. Moreover, the binding materials, which are often soluble polymers, tend to form viscous gels upon dissolution, which further reduces the tablet disintegration and finally the API release (15).

Immediate-release formulations are often desired to obtain a fast onset of the therapeutic effect of the API. Disintegration of these tablets is the first step before the drug dissolves and therefore of fundamental importance to achieve bioavailability and a rapid onset of the therapeutic effect of the drug.

In general, disintegrants cause the tablet to break up, when it comes in contact with water and helps to overcome the cohesive forces in the compact. The resulting particle size and the available surface generated after disintegration enhances the dissolution rate of the API. Early disintegrants included starch- and cellulose-based excipients, such as natural starch, pregelatinized starch, microcrystalline cellulose, and low-substituted hydroxypropyl cellulose. Due to chemical modifications, which decreased solubility and increased hydrophilicity at increased swelling, more effective disintegrants were developed, so-called superdisintegrants. They can be classified in chemically modified cellulose (croscarmellose sodium (CCS)), chemically modified starches (sodium starch glycolate (SSG)), and in pure synthesized copolymers, like crospovidone (CPV). Disintegrants act via different mechanism (e.g. swelling or shape recovery). In the case of swelling, the disintegrant absorbs water from the surrounding medium. This swelling results in multidirectional disruption of the tablets or granules structure during the disintegration process. The effectiveness of the disintegrant is dependent on the swelling extent and the swelling force generated during the water uptake. In the case of shape recovery, particles that are deformed, e.g. by the tableting process, regain their original shape, if water penetrates into the tablets, resulting on a unidirectional deformation of the tablet matrix.

Disintegration is often concentration dependent, resulting in an optimal concentration to achieve the best disintegration effect (16, 17). At the same time, the effect of disintegrants is often particle size and particle shape dependent (18–20). In wet granulation, the inclusion of the disintegrant in both intra-granular and extra-granular phase is often recommended, since the generated dispersion was reported to be finer, thereby increasing the available surface area (21, 22).

This is especially important, when high drug concentrations with a poor solubility are in focus (23). However, depending on the formulation used, the disintegration was reported to be faster when the disintegrant was added extra-granular only (24).

When disintegrants are added intra-granular prior to the melt granulation step, temperature and shear during the granulation process might influence the disintegrant performance. Until now, no systematic evaluation of different superdisintegrants exists concerning their suitability to be added to the melt granulation process.

The current study sought to examine the need of disintegrants to obtain immediate-release tablets prepared from twin-screw melt granules.

In this study, the three different superdisintegrants (CCS; CPV; SSG), with a comparable mean particle size of 40 μm, were tested in three weight fractions (4, 6, and 8% (w/w)). Moreover, in the case of the 6% (w/w) formulation, varying portions of this amount were added intra-granular. The effect on tabletability, friability, disintegration, and drug dissolution was tested on a model formulation of paracetamol (PCM) and polyethylene glycol 6000 (PEG) as a melt binder.

As model excipient, the highly soluble drug paracetamol (PCM) was chosen to show process-related influences on the disintegration and drug dissolution, rather than effects that are related to poor wettability or poor solubility of the drug itself. PCM has no lack in solubility (23.7 mg/ml at 37°C), its solubility is not pH-dependent below pH 9, and it belongs to the biopharmaceutical classification system (BCS) class III (25).

## MATERIAL AND METHODS

### Material

PCM (d_50_ = 7.6 μm (laser diffraction, Helos KF (Sympatec GmbH, Clausthal-Zellerfeld, Germany) dry-dispersed with a pressure of 1 bar, focal length 200 mm, calculated according to Frauenhofer theory, software Windox 4.2.1.) was obtained from Mallinckrodt Pharmaceuticals (Staines-Upon-Thames, UK). Due to its low melting point (55–60°C), the binder PEG 6000 was used and purchased from Carl Roth (Karlsruhe, Germany). Granulation of PCM required the addition of 1% (w/w) colloidal silicium dioxide (AEROSIL® 200) which was a gift from Evonik (Evonik Resource Efficiency GmbH, Hanau-Wolfgang, Germany). Sodium starch glycolate (Vivastar® P) and croscarmellose sodium (Vivasol® GF) were donated by JRS (J. RETTENMAIER & SÖHNE GmbH + Co KG, Rosenberg, Germany). Additionally, crospovidone (Kollidon® CL-F) was tested as a disintegrant, which was a gift from BASF (BASF SE, Ludwigshafen, Germany). Magnesium stearate (Ligamed® MF-2-V) was donated by Peter Greven (Peter Greven GmbH & Co. KG, Bad Münstereifel, Germany).

### Methods

#### Physical Mixtures (PM)

PCM was formulated with 10% (w/w) PEG as binding material. PCM required the addition of 1% (w/w) colloidal silicium dioxide (SDO) to enable feeding of the material into the extruder. PMs (batch size: 150 g) were prepared by using a Turbula blender (Willy A. Bachofen AG Maschinenfabrik, Switzerland), rotating at 50 rpm for 10 min. The obtained PMs were further used for twin-screw melt granulation (TSMG). Depending on the formulation, disintegrants were added proportional in the mixture. Short names used in text and graphics of this study are given in Table I (CCS), Table II (CPV), and Table III (SSG). The extra-granular components (disintegrant and magnesium stearate (MGST)) were added after granulation. Disintegrants were added extra-granular and mixed for another 5 min using a Turbula blender (Willy A. Bachofen AG Maschinenfabrik, Switzerland), rotating at 50 rpm. MGST was added in the second step and mixed for further 30 s.

**Table I:** Short Names of the Croscarmellose Sodium (CCS) Formulations (% w/w)

Short name	Intra-granular	Extra-granular
Gr. PCM 10% PEG	Granules PCM 88.11% +9.9% PEG +0.99% SDO	1% Mgst
CCS 4%	Granules PCM 84.55% +9.5% PEG +0.95% SDO	1% Mgst +4% CCS
CCS 6%	Granules PCM 82.77% +9.3% PEG +0.93% SDO	1% Mgst +6% CCS
CCS 6% SDO 1%	Granules PCM 81.88% +9.2% PEG +0.92% SDO	1% Mgst + 6% CCS +1% SDO
CCS 8%	Granules PCM 80.99% +9.1% PEG +0.91% SDO	1% Mgst +8% CCS
CCS 6% 20% intern	Granules PCM 82.77% +9.3% PEG +0.93% SDO+1.2% CCS	1% Mgst +4.8% CCS
CCS 6% 40% intern	Granules PCM 82.77% +9.3% PEG +0.93% SDO+2.4% CCS	1% Mgst +3.6% CCS
CCS 6% 60% intern	Granules PCM 82.77% +9.3% PEG +0.93% SDO+3.6% CCS	1% Mgst +2.4% CCS
CCS 6% 80% intern	Granules PCM 82.77% +9.3% PEG +0.93% SDO+4.8% CCS	1% Mgst +1.2% CCS
CCS 6% 100% intern	Granules PCM 82.77% +9.3% PEG +0.93% SDO+6% CCS	1% Mgst

*CCS* croscarmellose sodium, *Mgst* magnesium stearate, *PCM* paracetamol, *PEG* polyethylene glycol, *SDO* colloidal silicium dioxide

**Table II:** Short Names of the Crospovidone (CPV) Formulations (% w/w)

Short name	Intra-granular	Extra-granular
Gr. PCM 10% PEG	Granules PCM 88.11% +9.9% PEG +0.99% SDO	1% Mgst
CPV 4%	Granules PCM 84.55% +9.5% PEG +0.95% SDO	1% Mgst +4% CPV
CPV 6%	Granules PCM 82.77% +9.3% PEG +0.93% SDO	1% Mgst +6% CPV
CPV 6% SDO 1%	Granules PCM 81.88% +9.2% PEG +0.92% SDO	1% Mgst +6% CPV +1% SDO
CPV 8%	Granules PCM 80.99% +9.1% PEG +0.91% SDO	1% Mgst +8% CPV
CPV 6% 20% intern	Granules PCM 82.77% +9.3% PEG +0.93% SDO+1.2% CPV	1% Mgst +4.8% CPV
CPV 6% 40% intern	Granules PCM 82.77% +9.3% PEG +0.93% SDO+2.4% CPV	1% Mgst +3.6% CPV
CPV 6% 60% intern	Granules PCM 82.77% +9.3% PEG +0.93% SDO+3.6% CPV	1% Mgst +2.4% CPV
CPV 6% 80% intern	Granules PCM 82.77% +9.3% PEG +0.93% SDO+4.8% CPV	1% Mgst +1.2% CPV
CPV 6% 100% intern	Granules PCM 82.77% +9.3% PEG +0.93% SDO+6% CPV	1% Mgst

*CPV* crospovidone, *Mgst* magnesium stearate, *PCM* paracetamol, *PEG* polyethylene glycol, *SDO* colloidal silicium dioxide

**Table III:** Short Names of the Sodium Starch Glycolate (SSG) Formulations (% w/w)

Short name	Intra-granular	Extra-granular
Gr. PCM 10% PEG	Granules PCM 88.11% +9.9% PEG +0.99% SDO	1% Mgst
SSG 4%	Granules PCM 84.55% +9.5% PEG +0.95% SDO	1% Mgst +4% SSG
SSG 6%	Granules PCM 82.77% +9.3% PEG +0.93% SDO	1% Mgst +6% SSG
SSG 6% SDO 1%	Granules PCM 81.88% +9.2% PEG +0.92% SDO	1% Mgst +6% SSG +1% SDO
SSG 8%	Granules PCM 80.99% +9.1% PEG +0.91% SDO	1% Mgst +8% SSG
SSG 6% 20% intern	Granules PCM 82.77% +9.3% PEG +0.93% SDO+1.2% SSG	1% Mgst +4.8% SSG
SSG 6% 40% intern	Granules PCM 82.77% +9.3% PEG +0.93% SDO+2.4% SSG	1% Mgst +3.6% SSG
SSG 6% 60% intern	Granules PCM 82.77% +9.3% PEG +0.93% SDO+3.6% SSG	1% Mgst +2.4% SSG
SSG 6% 80% intern	Granules PCM 82.77% +9.3% PEG +0.93% SDO+4.8% SSG	1% Mgst +1.2% SSG
SSG 6% 100% intern	Granules PCM 82.77% +9.3% PEG +0.93% SDO+6% SSG	1% Mgst

*Mgst* magnesium stearate, *PCM* paracetamol, *PEG* polyethylene glycol, *SDO* colloidal silicium dioxide, *SSG* sodium starch glycolate

#### Twin-Screw Melt Granulation (TSMG)

TSMG (batch size: 150 g) was performed using a co-rotating twin-screw extruder (ZE12, Three-Tec GmbH, Seon, Switzerland) with a functional length of 25:1 L/D (length/diameter) and a 12 mm screw diameter (screw configuration: supplementary data: Fig. S1). The extruder barrel consisted of five individually adjustable heating zones to ensure sufficient melting and distribution of the binder. The process temperature was set up to 95°C in the high shear region of the extruder screws (30°, 60°, 60°, and 90° 18-mm-4-disc-kneading elements).

At the terminal zone of the barrel, the temperature was reduced to 75°C to allow solidification of the material. During melt granulation, the screw speed was set to 100 rpm, and no die plate was mounted at the end of the extruder barrel. A volumetric feeder system ZD9 (Three-Tec GmbH, Seon, Switzerland) was used to enable a constant feed rate of 0.1 kg/h. Granules obtained were further dry sieved using a 1 mm sieve in an Erweka wet granulator FGS with an AR 402 drive unit (Erweka, GmbH, Heusenstamm, Germany) prior to compaction.

#### Investigation of the Disintegrants

##### Particle Size Distribution

The particle size distribution (n=3) of the disintegrants was measured with a laser diffraction (λ= 655 nm) particle size analyser (Horiba LA-920, Horiba Ltd, Japan). A dry dispersion method was used with a pressure of 3 bar. The SPAN represents the width of the PSD and is calculated using Eq. (1):

1 $$ SPAN=\frac{d_{90}-{d}_{10}}{d_{50}} $$

##### Scanning Electron Microscopy (SEM)

Images of the disintegrants were taken using a scanning electron microscope (SU 3500, Hitachi High Technologies, Krefeld, Germany). The samples were mounted with a double adhesive photo sticker and coated with a thin layer of gold using a sputter coater for 2 min at 1.7 kV (Polaron SC7640, Quorum Technologies Ltd, Lewes, UK). Images were captured at an acceleration voltage of 10 kV in high vacuum mode and a secondary electron detector.

#### Density

Pycnometric density was measured using the AccuPyc 1330 helium pycnometer (Micromeritics GmbH, Aachen, Germany). The chamber was purged 20 cycles prior to analysis. A fill pressure of 136.86 kPa and an equilibration rate of 0.0345 kPa/min were used for measurements. The cycle was repeated up to 25 times or until a standard deviation of 0.01% was reached. The density was employed for the calculation of the SF.

#### Compaction Studies

The tablets (n=5) were compressed on a single punch tablet press (StylOne Classic 105 ML, Medelpharm, Beynost, France/Romaco Kilian, Cologne, Germany) with a 8 mm flat face tooling. Five levels of compaction pressures from 50 to 250 MPa were applied at constant tableting speed (dwell-time: 6–7 ms; compression time on average: 110 ms). The die was filled manually.

#### Out-of-Die Analysis

After 24 h of storage, the tablets were analysed by means of their tablet weight (analytical balance, AG 204, Mettler Toledo GmbH, Gießen, Germany), height (Mitutoyo Absolute ID C125B, Mitutoyo Deutschland GmbH, Neuss, Germany), diameter, and crushing strength (Erweka TBH 210, Erweka GmbH, Heusenstamm, Germany). The tensile strength (TS; Eq. (2)) and the solid fraction (SF; Eqs. (3), (4), and (5)) were calculated from the obtained data. TS (Eq. (2)) is the tablet crushing strength normalized by the dimension of the tablet and it is therefore independent of its geometry (26):

2 $$ TS=\frac{2F}{\pi dh} $$

where *F* is the crushing strength, *d* the diameter, and *h* the thickness of the tablet.

SF (Eqs. (3)–(5)) represents the apparent density (*P*_*app*_) of the compact calculated from the tablet weight (*m*) and its volume relative to the pycnometric density (*P*_*pyc*_) of the powder.

3 $$ SF=\frac{P_{app}}{P_{pyc}} $$

4 $$ {P}_{app}=\frac{m}{V_p} $$

5 $$ {V}_p=\pi \ast {\left(\frac{d}{2}\right)}^2\ast h $$

In Eq. (5), *Vp* is the volume of the compact calculated based on *d and h*, which describe the tablet diameter and thickness.

#### Production of Tablets for Further Testing

The tablets for friability, disintegration, and dissolution studies were produced with a clinical relevant concentration of 500 mg PCM. Tablets were compressed with a compaction simulator (StylOne Classic 105 ML, Medelpharm, Beynost, France/Romaco Kilian, Cologne, Germany) and a 13 mm round tooling. A compaction pressure of 150 MPa was applied at constant tableting speed (dwell-time: 6–7 ms; compression time on average: 110 ms).

#### Friability Test

The friability of the tablets was tested according to Ph. Eur. 2.9.7 (27): Friability of uncoated tablets. Approx.: 6.5 g of tablets (removed from dust and accurately weighed) was placed into a friability tester (Erweka TA3R, Erweka GmbH, Heusenstamm, Germany), which operated at 25 ± 1 rpm. After 100 rotations, the dust was removed, and the tablets were weighed using an analytical balance (AG 204, Mettler Toledo GmbH, Gießen, Germany). According to Ph. Eur. 2.9.7, a maximum loss of mass not greater than 1.0% is acceptable (27).

#### Disintegration Test

The disintegration test (n=6) was performed according to Ph. Eur. 2.9.1. and the monograph of uncoated tablets (28). The disintegration time of the tablets (n=6) was determined in 800 ml demineralized water (37 ±1 °C) using an automatic disintegration tester, according to test A of the Ph. Eur. (Erweka ZT72, Erweka GmbH, Heusenstamm, Germany). To meet the requirements, the uncoated tablets should disintegrate within 15 min (= 900 s) (28).

#### Dissolution Test

Dissolution tests (n=6) were performed using a USP Dissolution Apparatus 2 (paddle method) (Sotax AT7, Sotax GmbH, Lörrach, Germany) with a rotation speed of 50 rpm. Analysis was performed according to the USP monograph for acetaminophen tablets with 900 ml of phosphate buffer at a pH of 5.8 ±0.1 and a set temperature of 37.0°C ±0.5°C. Tablets (n=6) containing 500 mg PCM were used for each experiment. For quantitative analysis, an Agilent 8453 in-line UV-VIS spectrophotometer (Agilent Technologies, Waldbronn, Germany) was used. Absorption was determined at a fixed wavelength of 260 nm. Dissolution tests were performed over a maximum period of 6 h with a maximum interval of 10 min between the measurements. Dissolution results were compared using Q_80%_, giving the time point at which 80% of the formulation is released. The USP 41 requires minimum of 30 min for the 80% dissolution of the “acetaminophen tablets” in phosphate buffer (pH = 5.8) (29), whereas in the Ph. Eur. 5.17.1, 45 min or less for the release of 80% API is reported (for conventional release dosage forms) (27).

## RESULTS

### Investigation of the Disintegrants

All disintegrants had a comparable mean particle size of 40 μm (Table IV). The particle size of CPV was broader compared to the particle size of CCS and SSG. This is indicated by the higher SPAN of 2.30 for CPV.

**Table IV:** Particle Size Distribution of the Disintegrants (Laser Diffraction, Dry Dispersion, 3bar)

Disintegrant	d_10_ [μm]	d_50_ [μm]	d_90_ [μm]	SPAN
CCS	23.1 ±0.1	39.7 ±0.1	69.0 ±0.4	1.16
CPV	17.0 ±0.5	40.4 ±0.4	109.7 ±1.3	2.30
SSG	23.2 ±0.3	39.9 ±0.3	66.3 ±0.2	1.08

*CCS* croscarmellose sodium, *CPV* crospovidone, *SSG* sodium starch glycolate

**Fig. 1:**
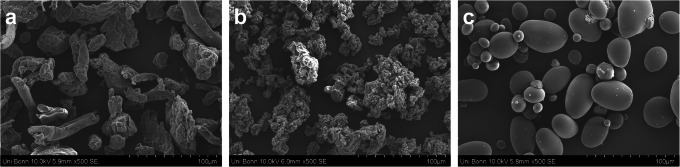
Scanning electron images of the disintegrants: **a** croscarmellose sodium (CCS); **b** crospovidone (CPV); **c** sodium starch glycolate (SSG)

SEM images determined similar particle size distribution of the disintegrants but a different morphology and shape. CCS (Fig. 1a) had a more elongated and fibre-like structure, whereas CPV showed a “popcorn” structure with a high porosity (Fig. 1b). SSG consisted of round particles with a very smooth surface structure (Fig. 1c).

### Density

Densities of the formulations under investigation are given in Table V. The values were used for the calculation of the SF of the tablets.

**Table V:** Pycnometric Densities of the Formulations

Formulation	Pycnometric density [g/cm^3^]	Formulation	Pycnometric density [g/cm^3^]	Formulation	Pycnometric density [g/cm^3^]
**Gr. PCM 10% PEG**			1.3050 ±0.0034		
**CCS 4%**	1.3094 ±0.0012	**CPV 4%**	1.3136 ±0.0022	**SSG 4%**	1.3158 ±0.0005
**CCS 6%**	1.3158 ±0.0011	**CPV 6%**	1.3452 ±0.0049	**SSG 6%**	1.3275 ±0.0006
**CCS 6% SDO 1%**	1.3203 ±0.0003	**CPV 6% SDO 1%**	1.3443 ±0.0004	**SSG 6% SDO 1%**	1.3378 ±0.0004
**CCS 8%**	1.3194 ±0.0003	**CPV 8%**	1.3684 ±0.0005	**SSG 8%**	1.3331 ±0.0004
**CCS 6% 20% intern**	1.3222 ±0.0021	**CPV 6% 20% intern**	1.3245 ±0.0004	**SSG 6% 20% intern**	1.3284 ±0.0005
**CCS 6% 40% intern**	1.3262 ±0.0000	**CPV 6% 40% intern**	1.3295 ±0.0006	**SSG 6% 40% intern**	1.3247 ±0.0032
**CCS 6% 60% intern**	1.3205 ±0.0010	**CPV 6% 60% intern**	1.3183 ±0.0019	**SSG 6% 60% intern**	1.3323 ±0.0006
**CCS 6% 80% intern**	1.3262 ±0.0004	**CPV 6% 80% intern**	1.3273 ±0.0034	**SSG 6% 80% intern**	1.3245 ±0.0005
.**CCS 6% 100% intern**	1.3217 ±0.0019	**CPV 6% 100% intern**	1.3443 ±0.0031	**SSG 6% 100% intern**	1.3270 ±0.0004

*CCS* croscarmellose sodium, *CPV* crospovidone, *Mgst* magnesium stearate, *PCM* paracetamol, *PEG* polyethylene glycol, *SDO* colloidal silicium dioxide, *SSG* sodium starch glycolate

### Compaction Studies

#### Tabletability

**Fig. 2:**
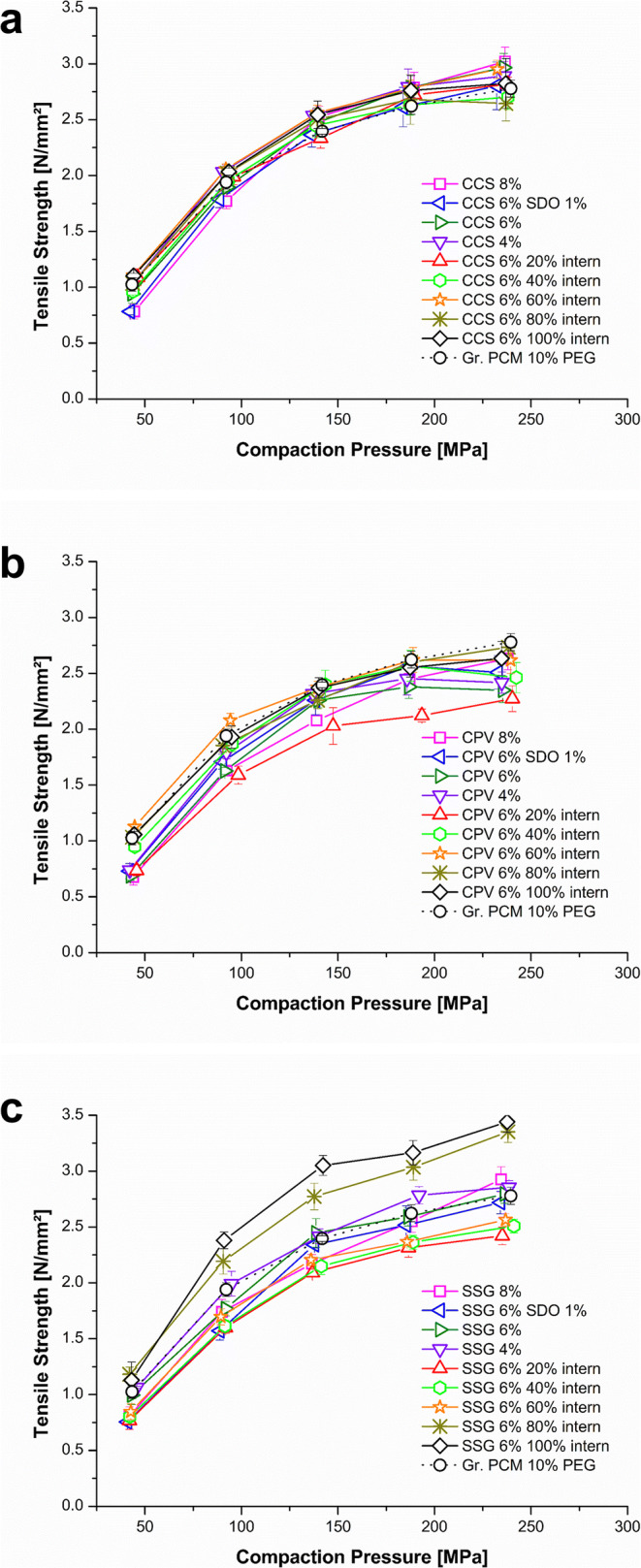
Tabletability plot of the formulations: **a** croscarmellose sodium (CCS); **b** crospovidone (CPV); **c** sodium starch glycolate (SSG)

Figure 2a shows the tabletability studies of the croscarmellose sodium (CCS) formulations. In general, the addition of CCS in the formulation had no impact on the tabletability (e.g. Gr. PCM 10% PEG: 2.40 ±0.03 N/mm^2^ at 150 MPa *vs.* CCS 8%: 2.53 ±0.10 N/mm^2^ at 150 MPa). In the case of using crospovidone (CPV), the addition of the disintegrant can slightly lower tensile strength (TS). This was pronounced, when high weight fractions of CPV were added extra-granular: CPV 8% (2.08 ±0.05 N/mm^2^ at 150 MPa); 6% (2.26 ±0.07 N/mm^2^ at 150 MPa); and 6% 20% intern (2.03 ±0.16 N/mm^2^ at 150 MPa) (Fig. 2b). Figure 2c shows the tabletability of the sodium starch glycolate (SSG) formulations in which a high weight fraction of the disintegrant intra-granular had a positive effect on the TS (SSG 6% 80% (2.77 ±0.12 N/mm^2^ at 150 MPa) and 100% intern (3.05 ±0.09 N/mm^2^ at 150 MPa)). When high weight fractions of SSG were added extra-granular (SSG 6% 20% intern (2.09 ±0.03 N/mm^2^ at 150 MPa), SSG 6% 40% intern (2.15 ±0.08 N/mm^2^ at 150 MPa), and SSG 6% 60% intern (2.21 ±0.05 N/mm^2^ at 150 MPa)), TS was slightly lowered. However, the effect was not pronounced for the SSG 6% (2.45 ±0.13 N/mm^2^ at 150 MPa) extra-granular, indicating that the effect was not concentration dependent.

#### Compactibility

**Fig. 3:**
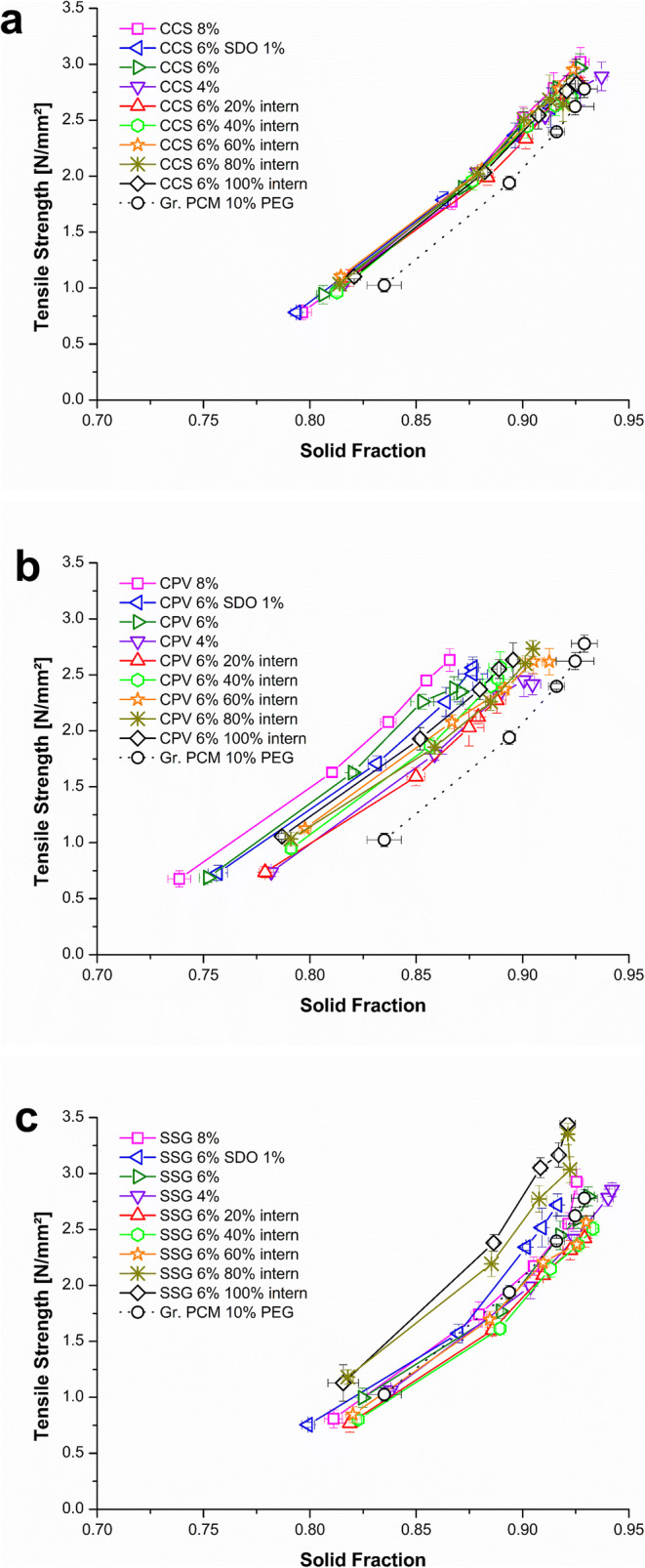
Compactibility plot of the formulations: **a** croscarmellose sodium (CCS); **b** crospovidone (CPV); **c** sodium starch glycolate (SSG)

In Fig. 3a, the compactibility plot of the CCS formulations are shown. It can be seen that the formulations containing CCS showed lower SF at comparable TS than the formulation without disintegrant (Gr. PCM 10% PEG). The shift to lower SFs was independent of the CCS concentration and the way of addition (extra- or intra-granular). Using CPV (Fig. 3b) as disintegrant, the effect on lowering the SFs of the tablets was higher compared to the CCS formulations (Fig. 3a). Being added extra-granular, CPV lowered TS and the SF of the formulation as a function of increased CPV weight fraction (4; 6; and 8%). The intra-granular addition of CPV compensated this effect concentration dependent. Regarding the 6% (w/w) formulations, the higher the intra-granular amount of CPV, the higher the SFs of the tablets. These formulations showed higher SFs and TS but still lower than the Gr. PCM 10% PEG, which contained no disintegrant. The effect of SSG addition on the SF was minor (Fig. 3c).

However, using SSG intra-granular (SSG 6% 80% intern and 100% intern) showed a higher bonding capacity than the formulation without disintegrant (Gr. PCM 10% PEG) and the formulations with high amounts of SSG extra-granular.

#### Compressibility

**Fig. 4:**
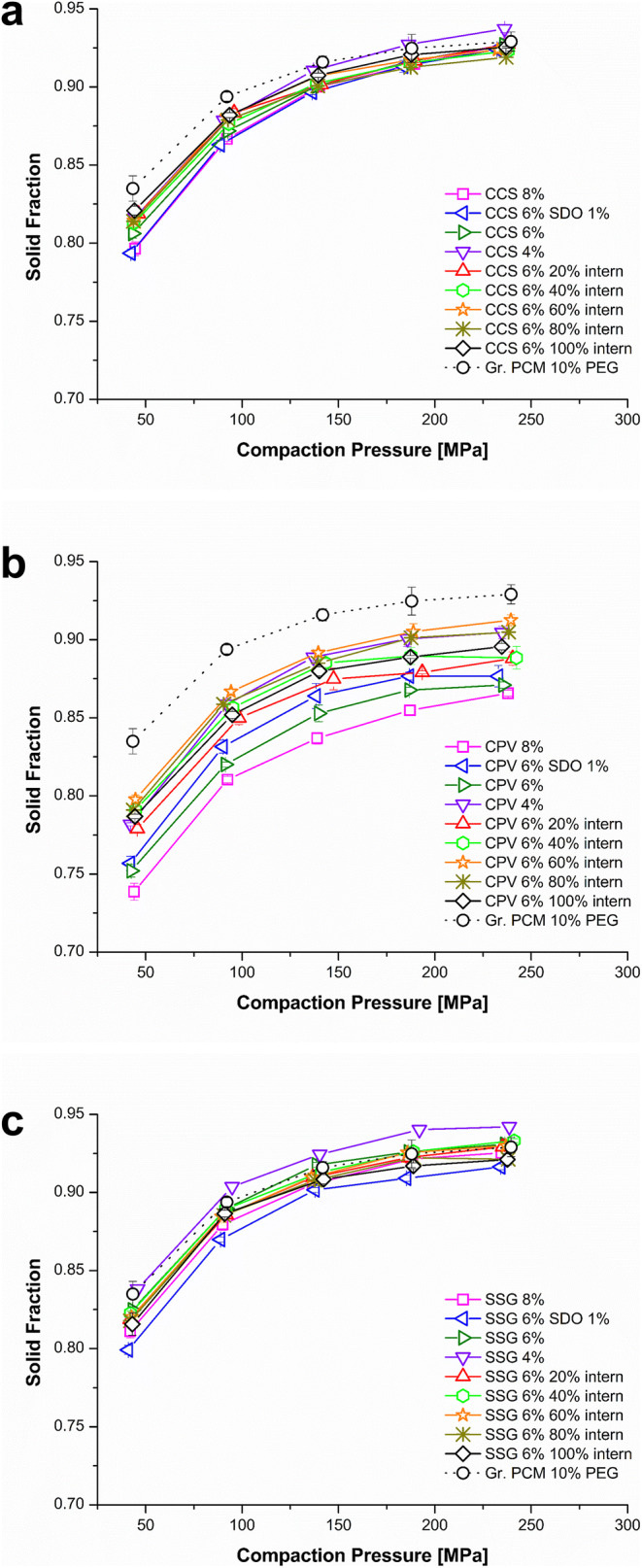
Compressibility plot of the formulations: **a** croscarmellose sodium (CCS); **b** crospovidone (CPV); **c** sodium starch glycolate (SSG)

All CCS formulations showed a weight fraction independent slightly lower SF than the Gr. PCM 10% PEG formulation (e.g. Gr. PCM 10% PEG: 0.92 ±0.01 at 150 MPa *vs.* CCS 8%: 0.90 ±0.01 at 150 MPa) (Fig. 4a). Figure 4b depicts the compressibility plots of the CPV formulations. In comparison to CCS, a higher effect on the SF can be seen. Moreover, the effect of CPV was weight fraction dependent (CPV 4% (0.89 ±0.01), CPV 6% (0.85 ±0.01), CPV 8% (0.84 ±0.01)). When adding CPV in high concentrations intra-granular, the effect was minor (CPV 6% 60% intern (0.91 ±0.01), CPV 6% 80% intern (0.90 ±0.01), and CPV 6% 100% intern (0.91 ±0.01)). In the case of the SSG formulations (Fig. 4c), the SFs were less affected by the disintegrant addition.

### Friability

In general, friability studies confirmed the results obtained from the tabletability studies (Table VI). All tablets produced with CCS showed acceptable friability (<1%) without any trend. The highest friability was obtained at a weight fraction of CCS 4% (0.47%), whereas the lowest friability occurred at CCS 6% and 6% 20% intern (0.22%), indicating that CCS has no positive or negative effect on the abrasiveness of the tablets.

**Table VI:** Friability [%] of the Formulations Under Investigation

Formulation	Gr. PCM 10% PEG [%]		
	0.22		
	CCS [%]	CPV [%]	SSG [%]
4%	0.47	0.27	0.28
6%	0.22	0.26	0.35
8%	0.31	0.29	0.43
6% +1% SDO	0.35	0.13	0.45
20% intern	0.27	0.38	0.32
40% intern	0.33	0.26	0.32
60% intern	0.22	0.29	0.30
80% intern	0.25	0.27	0.28
100% intern	0.23	0.16	0.26

*CCS* croscarmellose sodium, *CPV* crospovidone, *Mgst* magnesium stearate, *PCM* paracetamol, *PEG* polyethylene glycol, *SDO* colloidal silicium dioxide, *SSG* sodium starch glycolate

Concerning their friability, all tablets produced with CPV met the requirements of the Ph. Eur. No clear trend among the different weight fractions of CPV was observed, which verified the results obtained from the compression studies. The lowest friability was measured for the tablets containing 6% CPV and 1% SDO (0.13%) extra-granular, followed by the tablets with 6% intra-granular (0.16%), indicating no influence on tablets friability.

Overall, the friability of the tablets produced with SSG was slightly higher than the friability of the tablets produced from the granules without any disintegrant (Gr. PCM 10% PEG: 0.22%). In addition, an increasing friability as a function of SSG weight fraction was obvious (SSG 4%: 0.28% *vs.* SSG 8%: 0.43%). The SSG intra-granular addition decreased the effect again, towards that of the tablets without disintegrant (SSG 6% 100% intern: 0.26% *vs.* Gr. PCM 10% PEG: 0.22%). However, the effect might be not relevant for commercial tablet production, since all tablets met the requirements of the Ph. Eur., as they showed a friability less than 1% (w/w).

### Disintegration

According to Ph. Eur. 2.9.1., uncoated tablets must disintegrate within 15 min, which is equal to a time of 900 s. Tablets produced without disintegrants are disintegrated within 30 min (1775 ±327 s), indicating that these tablets did not meet the requirements for an immediate-release formulation. Using CCS as disintegrant, all formulations under investigation showed an acceptable disintegration time (Table VII). No clear trend can be seen between the different formulations using CCS. A clear trend can be seen for the CPV formulations. CPV reduced the disintegration time more effectively than CCS, especially when incorporated extra-granular prior tableting (CPV 8%: 78 ±7 s *vs.* CCS 8%: 390 ± 93 s). The disintegration time decreased with increasing CPV content. However, the addition of CPV intra-granular resulted in increasing disintegration time. The higher the integrated amount, the slower the disintegration process. Tablets using 100% CPV intra-granular (957 ± 97 s) did not disintegrate within 15 min and failed the requirements of the Ph. Eur. 2.9.1.

**Table VII:** Disintegration Times [s] of the Formulations Under Investigation

Formulation	Gr. PCM 10% PEG [s ±SD]		
	1775 ±327		
	CCS [s ±SD]	CPV [s ±SD]	SSG [s ±SD]
4%	435 ±69	211 ±27	598 ±37
6%	360 ±88	129 ±29	492 ±67
8%	390 ±93	78 ±7	513 ±38
6% +1% SDO	307 ±109	109 ±15	471 ±27
20% intern	237 ±63	127 ±11	319 ±50
40% intern	249 ±46	257 ±71	513 ±44
60% intern	325 ±67	299 ±40	559 ±56
80% intern	287 ±45	407 ±36	532 ±33
100% intern	434 ±70	957 ±97	553 ±68

*CCS* croscarmellose sodium, *CPV* crospovidone, *Mgst* magnesium stearate, *PCM* paracetamol, *PEG* polyethylene glycol, *SDO* colloidal silicium dioxide, *SSG* sodium starch glycolate

Overall, formulations using SSG showed the longest disintegration times (SSG 8%: 513 ±38 s). SSG 6% 20% intern (319 ±50 s) exhibited lower disintegration time, than the other formulations. No clear trend could be observed concerning the disintegration efficiency, as it was seen for CPV (Table VII).

### Dissolution

To meet the USP, a Q=80% release within 30 min for PCM tablets is needed (29), whereas the Ph. Eur. 5.17.1 requires a dissolution within 45 min for “conventional release” dosage forms (27).

**Fig. 5:**
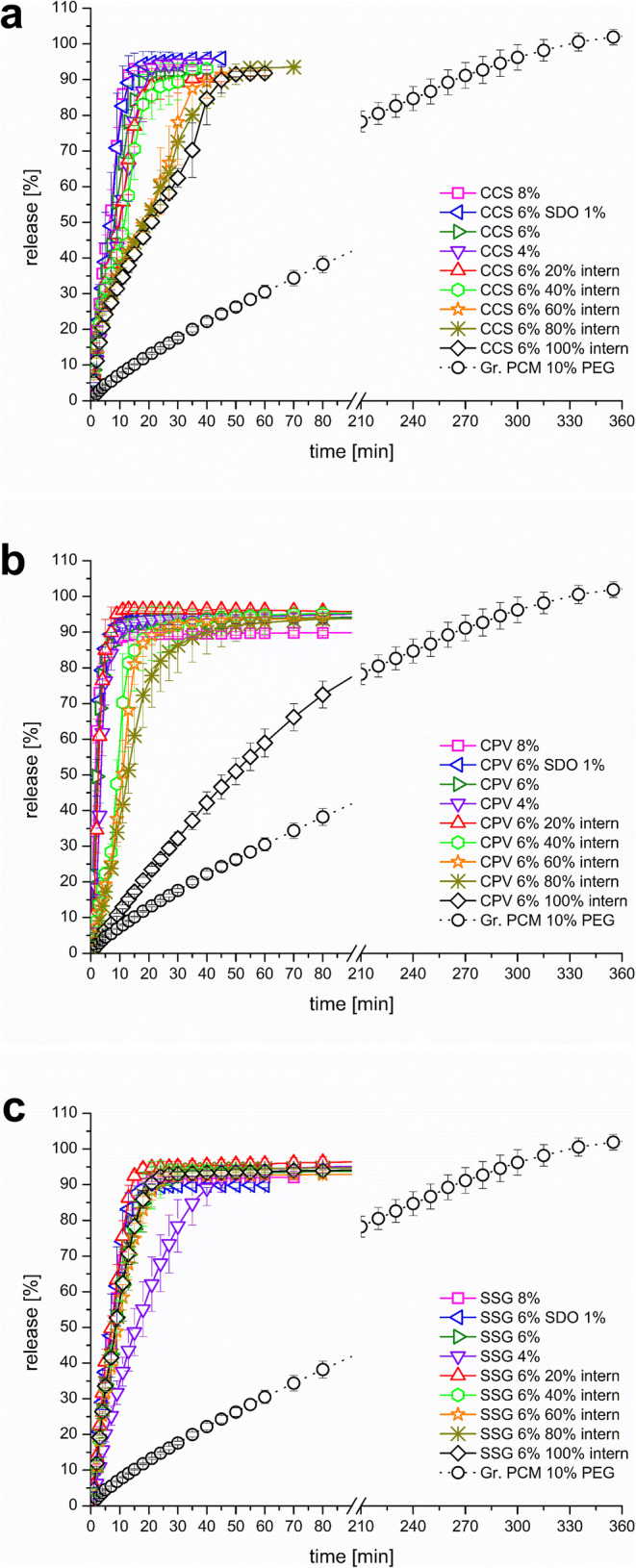
Dissolution of the formulations: **a** croscarmellose sodium (CCS); **b** crospovidone (CPV); **c** sodium starch glycolate (SSG)

Tablets without any disintegrant exhibited a slow dissolution, which followed a zero-order kinetic in the first hours (Fig. 5). A release of 80% was obtained after approx. 220 min, which does not meet the requirements of the USP and Ph. Eur. for a conventional release formulation or for PCM tablets.

**Fig. 6:**
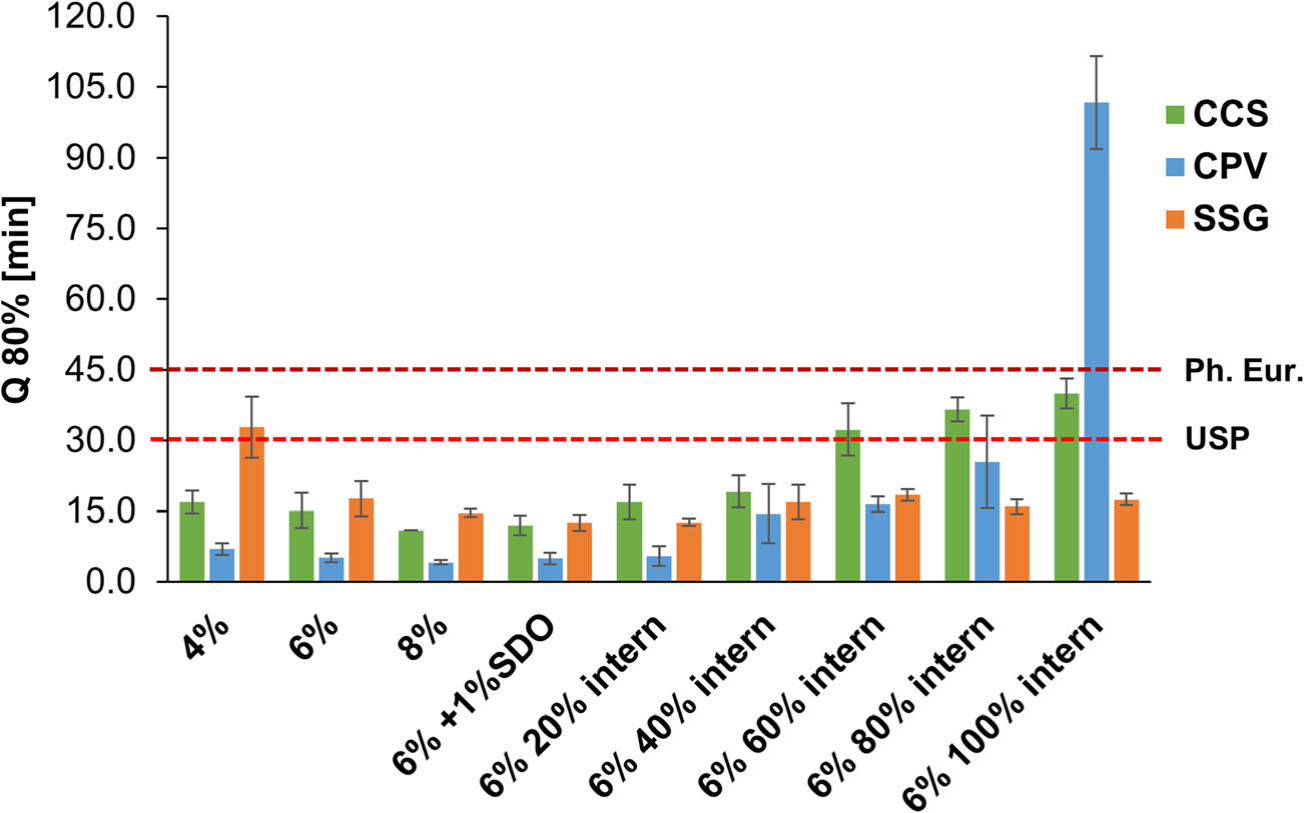
Q_80%_ of dissolution of the formulations under investigation

Figure 5a, b, and c present the obtained dissolution curves of the formulations under investigation. Figure 6 and Tab. S8 (supplementary data) show the Q_80%_ interval [min]. Regarding the CCS formulations, dissolution increased only slightly as a function of disintegrant content (Fig. 5a). In the case of the incorporation of CCS intra-granular, a significant delay was observed when more than half of the content of disintegrant was integrated. The Q_80%_ occurred later than 30 min, which is the required value for the USP. However, no significant difference can be seen between CCS 6% 60% intern (Q_80%=_ 32.3 ±5.5 min), CCS 6% 80% intern (Q_80%=_ 36.7 ±2.6 min), and CCS 6% 100% intern (Q_80%=_ 40.0 ±3.2 min).

In the case of CPV, the fastest release was obtained, when 8% CPV (Q_80%=_ 4.2 ±0.4 min) was integrated extra-granular (Fig. 5b). Also 4% CPV (Q_80%=_ 7.0 ±1.3 min) and 6% CPV (Q_80%=_ 5.2 ±1.0 min) extra-granular resulted in a very fast release, indicating the high potential of the disintegrant to enhance dissolution of the tablets. However, using CPV intra-granular delayed release, especially when more than 20% where integrated, tablets with 20% (Q_80%=_ 5.5 ±2.1 min), 40% (Q_80%=_ 14.5 ±6.3 min), and 60% (Q_80%=_ 16.5 ±1.6 min) of the 6% CPV intra-granular still met the requirements of the USP and tablets achieved more than 80% drug release within the first 30 min.

When 100% of CPV were granulated, CPV lost a remarkable proportion of its disintegration efficiency. Tablets of CPV 6% 100% intern achieved 80% release within more than 90 min (Q_80%=_ 101 ±9.8 min).

Using SSG resulted in the slowest dissolution profile considering the 4% extern formulations of the three disintegrants (Fig. 5c). The formulation SSG 4% extern (Q_80%=_ 32.8 ±6.5 min) did not meet the requirements of the USP. However, in the case of 6% (Q_80%=_ 17.7 ±3.7 min), results were comparable to those of CCS, but not as effective as CPV. Interestingly, the incorporation intra-granular of SSG does not result in any loss of the disintegrant efficiency, as it was seen for CCS and CPV.

Using 4% extra-granular, CPV (Q_80%=_ 7.0 ±1.3 min) performed more effective than CCS (Q_80%=_ 17.0 ±2.4 min), and CCS was more effective than SSG (Q_80%=_ 32.8 ±6.5 min). Using 6% extra-granular, CCS and SSG performed similar (Q_80%=_ 15.2 ±3.7 min and 17.7 ±3.7 min), whereas the 8% extra-granular CCS (Q_80%=_ 11.0 ±0.0 min) was more efficient than the respective SSG formulation (Q_80%=_ 14.7 ±0.8 min). In both cases (6% and 8% extra-granular), CPV was superior, showing very short time to achieve 80% release of only 5.2 ±1.0 min and 4.2 ± 0.4 min. Using 1% SDO as additional wicking agent had only a minor effect on the release rate and did not significantly impact the dissolution, when compared to the 6% disintegrant extra-granular. In the case of adding 20% of the 6% disintegrant intra-granular, Q_80%_ did not change compared to the 100% extra-granular formulation using CCS and CPV. For SSG, the 6% 20% intern formulation performed marginally faster than the SSG 6% formulation (Q_80%=_ 12.7 ±0.8 *vs.* 17.7 ±3.7). Similar relations were already seen in the disintegration results (Table VII). In the case of 40% intern formulations, the Q_80%_ increased for CPV (Q_80%=_ 14.5 ± 6.3 min), but for CCS and SSG, the data were similar to those of the 6% formulation containing the disintegrant extra-granular. Using CCS, Q_80%_ increased above the critical value of 30 min, using more than 60% intra-granular. In the case of CPV, using more than 80% intra-granular resulted in tablets that did not meet this requirement of the USP. Using 100% intra-granular resulted in tablets that achieved 80% release within more than 90 min, indicating noteworthy loss of the disintegration efficiency. SSG showed no remarkable loss of its disintegration efficiency when integrated into the granulation process.

## DISCUSSION

Results show that the disintegration and the dissolution of the tablets were less dependent on the disintegrants concentration but rather on the location of the disintegrant (extra- or intra-granular).

In general, the extra-granular disintegrant addition resulted in the fastest dissolution of the model compound PCM. Looking at the formulations with disintegrant extra-granular, the rank order of performance was CPV faster than CCS faster than SSG. Furthermore, the concentration needed of SSG was higher than that of CCS and CPV to meet the requirements of the USP.

Except for the SSG formulation, no difference between the 4 and 6% extra-granular formulations was seen. However, 8% CPV and CCS extern was more effective than using 4% CPV and CCS, but the effect might be irrelevant for the patient’s therapy.

In literature, the three superdisintegrants are often compared (30–35) and variations between different test methods (e.g. pH of the test medium) or different vendors (e.g. extent of crosslinking or the degree of substitution) might explain the unclear order of their effectiveness at similar weight fractions (20, 36, 37). Also the influence of particle size of different disintegrants has been shown by several working groups (32, 38–40). In this study disintegrants with comparable particle size were used.

The results obtained in this study might be explained by the different mechanisms of action of the disintegrants. Proposed mechanisms of disintegration are wicking, swelling, shape recovery and particle repulsion, which lead to a disruption of the physicochemical bonds in the tablets (41–43). Synergistic combination of the proposed mechanisms are possible (17). After contact with water, wettability and the penetration of water into the tablet are the first steps that need to be fulfilled, before the tablet disintegrates into smaller particles. Wicking is the proposed mechanism for microcrystalline cellulose (MCC), which describes the capillary action of the material and its potential to pull water into the tablet. Presumably wicking supports swelling and shape recovery by absorbing more water into the tablet, but does not active generate pressure to destroy particle bonds (44), similar effects might be achieved using low proportions of SDO. However, using even 1% of SDO did not show any significant effects on the formulations in this study.

CCS and SSG are known to act via swelling, which leads to a multidirectional volume expansion of the compact. However, the water uptake and swelling capacity of SSG is higher compared to CCS, whereas CPV shows only a low swelling capacity (36, 45). The authors described an enormous volume expansion of SSG. Recently it was proposed that CPV mainly acts via shape-recovery, which is a reversible viscoelastic process of deformation, which is activated after contact with water (46). The proposed mechanism was confirmed by high resolution real-time magnetic resonance imaging (47) and simple image analysis using a digital camera (48). In contrast to swelling, shape recovery results in a more unidirectional volume expansion of the tablet, in the opposite direction of compression, while releasing the energy, which is stored in the compact. The disintegrant particles are regaining their original shape, when they come into contact with water. This mechanism also explains that its dissolution efficiency increased with compaction force (49). The predominant mechanism described for CCS is wicking and swelling (35, 50). Disintegrants that act primary via wicking and swelling (CCS and SSG), show high capillary action and swelling. Moreover their disintegration time decreased, with increasing compaction pressure (46). Recently a change from predominant swelling mechanism to shape recovery was proposed for CCS depending on its concentration (48). This behaviour was explained with the formation of a hydrated gel matrix, maintaining tablets integrity at high concentrations. Concentration dependent decrease of disintegration time was also described from other authors (17, 51, 52). However, for the melt granulation process, we did not see any decrease in disintegration using higher disintegrant concentrations.

This might be attributed to the very low porosity of the tablets made from the TSMG in contrast to the study of Berardi et al. (48) and Ferrero et al. (52) and where tablets were made by direct compression. With the low porosity achieved via TSMG any volume expansion went into bond weakening and subsequent disintegration rather than gel formation, which would also explain the reached plateau in terms of disintegration and dissolution rather than an optimum.

In this study CCS and CPV showed a concentration dependent decrease in their disintegration efficiency (Tab. 7), when integrated intra-granular. The effect was more pronounced for CPV. Tablets using 100% CPV intra-granular did not disintegrate within 15 min, whereas tablets using 100% CCS still meet the requirements of the Ph.Eur. However, looking at the dissolution profiles, tablets with more than 40% of CCS intra-granular did not meet the requirements of the USP. This concentration dependent behaviour was not seen for the formulation produced with SSG.

The effect of extra- and intra-granular addition of disintegrant is diversely discussed in literature, mainly depending on the formulation characteristics, rather than on the disintegrant itself. Some authors found improved efficiency of the disintegrant when added intra-granular, whereas others found contrary results (21–24, 53, 54). Johnson et al. studied the effect of tablet formulation solubility and hygroscopicity on dissolution efficiency. The decrease in disintegration efficiency occurred in wet-granulated formulations containing highly soluble and or hygroscopic excipients (55). Similar results were shown by Gordon et al., who showed that hygroscopic ingredients can decrease the effectiveness of superdisintegrants (56). Both authors explained the decrease with a competitive inhibition of the disintegrant by the other tablet components competing for the locally available water. Their observations were recently confirmed for soluble fillers (57). The different behaviour of the disintegrant when in-cooperated in the granulation step might be again explainable with their different disintegration mechanism.

If the disintegrant is coated with a film of hydrophobic or slowly dissolving substance, disintegration might be negatively affected. This effect is well described for MGST, which can slow down disintegration or the dissolution rate, due to a hydrophobic surface coating. This effect was shown to be less pronounced for disintegrants that undergo intensive swelling like SSG (58, 59).

The omnipresent PEG in the tablet after the granulation process results in a viscous gel when coming into contact with water. This effect might explain a loss in dissolution efficiency of CPV and CCS.

The loss of disintegration efficiency, e.g. during wet-granulation process, was already described (30, 60), including the effect of recompression on disintegrant efficiency in tablets prepared by wet granulation (30). Very similar results were obtained for the disintegrants under investigation. Gould et al. 1985 showed that, all disintegrants placed intra-granular showed a loss of disintegration efficiency. Explotab® (SSG) retained good efficiency after rework. CPV act very sufficient when added before the second compression step extra-granular. The loss of the disintegration efficiency was explained by the authors with the different structure of the disintegrants. CPV shows a sponge-like matrix (popcorn structure), whereas CCS consist of “spaghetti-like” fibrous being broken down by the first compaction process.

Similar effects might explain the loss in disintegration and prolonged dissolution of the compacts in this study, as the TSMG process applies high shear energy to the product, the use of kneading elements might result in a destruction of the disintegrants structure, resulting in a loss of their efficiency. This hypothesis is supported by the results obtained from the compressibility studies. The formulations containing CPV extra-granular showed a lower SF (higher porosity) compared to the formulations containing CPV intra-granular. This might be explained by a change of the structure of CPV during granulation, negatively affecting its elastic recovery during tableting process and consequently the disintegration and the dissolution performance of the formulation. Interestingly the lower SF of the formulation with CPV did not influence the TS of the tablets in a large extent. This can be explained with high plasticity of the melt granules (61), maintaining the bonding capacity of the formulation.

In contrast to CPV, the majority of the SSG particles might be unaffected by the granulation process, therefore most of the enormous swelling capacity is maintained after the granulation process. Moreover a positive effect on the TS of the formulations containing high concentrations of SSG intra-granular was observed. The higher TS did not negatively influence the dissolution performance of the tablets.

However, it was postulated that the heat exposure during the melt granulation process, might result in an unwanted loss of disintegration efficiency, especially when using starch-based disintegrants. A pregelatinization might occur, negatively affecting the disintegration performance (62). In contrast to native starches, the used SSG was not influenced by the melt granulation process (T_max_: 95°C and absence of water) and maintained its disintegration capacity.

## CONCLUSION

The current study showed that especially extra-granular addition of superdisintegrant revealed fast disintegrating tablets with subsequently short Q_80%_. At the same time, the benefits of tablets from TSMG like excellent tensile strength and friability could be remained even in combination with a high drug load (> 80% (w/w)). The rank order in efficiency for extra-granular addition was CPV > CCS > SSG. For SSG, higher concentrations of >6% (w/w) were needed, whereas already 4% (w/w) achieved acceptable results for CPV and CCS. Intra-granular addition showed mainly negative effects on the disintegration performance. This effect was very pronounced, when CPV was added into the granulation process and to a lesser extent for CCS. In contrast, SSGs disintegration efficiency was unaffected by the granulation process. The results are of fundamental importance, when using TSMG in a continuous processing line and additional mixing steps after granulation should be avoided.

## Supplementary Information

High Resolution Image (TIF 2165 kb)

ESM 2 (DOCX 14 kb)

## List of Symbols and Abbreviations

API: active pharmaceutical ingredient
BCS: biopharmaceutical classification system
CCS: croscarmellose sodium
CPV: crospovidone
L/D: length/diameter
MCC: microcrystalline cellulose
MGST: magnesium stearate
PCM: paracetamol
PEG: polyethylene glycol
Ph. Eur.: European Pharmacopoeia
PM: physical mixture
PSD: particle size distribution
SDO: colloidal silicium dioxide
SEM: scanning electron microscope
SF: solid fraction
SSG: sodium starch glycolate
TS: tensile strength (N/mm^2^)
TSMG: twin-screw melt granulation
USP: United States Pharmacopeia
