# Supplementary figures and images for: Immediate-Release Formulations Produced via Twin-Screw Melt Granulation: Systematic Evaluation of the Addition of Disintegrants
Source: AAPS PharmSciTech. 2021 Jun 16;22(5):183. doi: 10.1208/s12249-021-02056-0 (PMC8208916; doi:10.1208/s12249-021-02056-0)

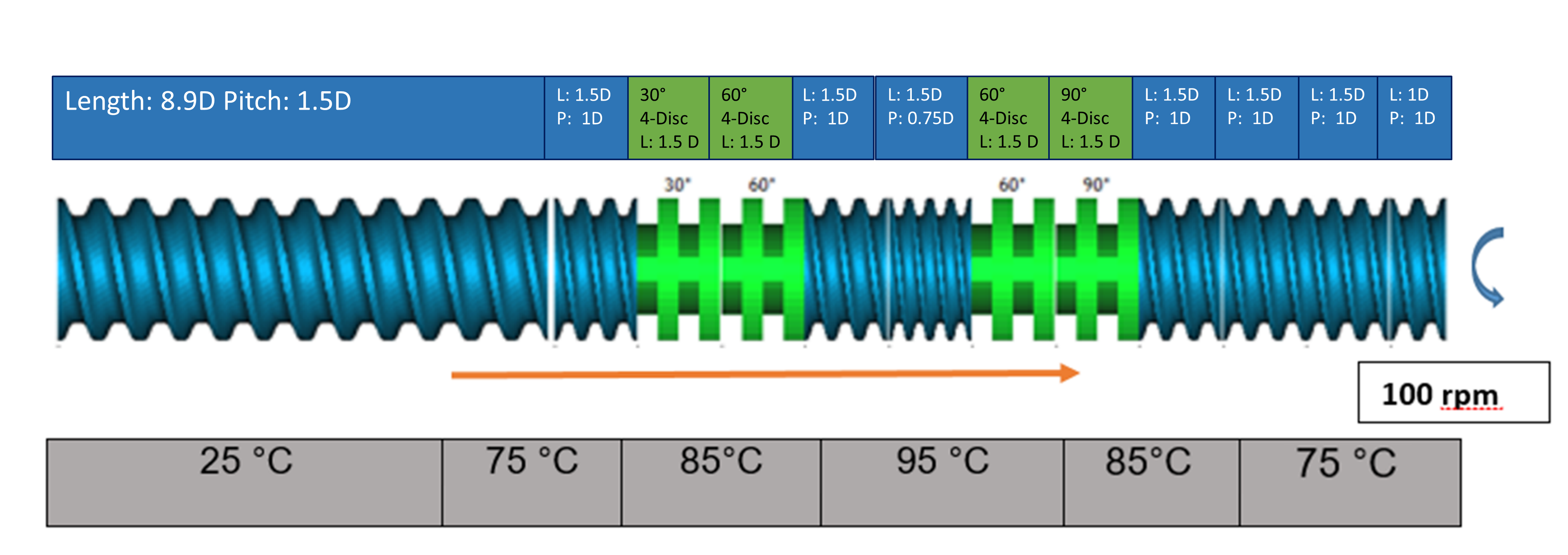

Supplement: Supplementary file 1 — High Resolution Image (TIF 2165 kb) [file 12249_2021_2056_MOESM1_ESM.tif]
