# Supplementary material for: Immediate-Release Formulations Produced via Twin-Screw Melt Granulation: Systematic Evaluation of the Addition of Disintegrants
Source: AAPS PharmSciTech. 2021 Jun 16;22(5):183. doi: 10.1208/s12249-021-02056-0 (PMC8208916; doi:10.1208/s12249-021-02056-0)
Supplement: Supplementary file 2 — (DOCX 14 kb) [file 12249_2021_2056_MOESM2_ESM.docx]

**Tab. S8:** Q_80%_ of dissolution of the formulations under investigation

| Formulation | Q_80%_ Gr. PCM 10% PEG [min ±SD] | | |
| --- | --- | --- | --- |
|  | 223.3 ±12.1 | | |
|  | **Q_80%_ CCS [min ±SD]** | **Q_80%_ CPV [min ±SD]** | **Q_80%_ SSG [min ±SD]** |
| 4 % | 17.0 ±2.4 | 7.0 ±1.3 | 32.8 ±6.5 |
| 6 % | 15.2 ±3.7 | 5.2 ±1.0 | 17.7 ±3.7 |
| 8 % | 11.0 ±0.0 | 4.2 ±0.4 | 14.7 ±0.8 |
| 6% +1% SDO | 12.0 ±2.1 | 5.0 ±1.2 | 12.6 ±1.7 |
| 20 % intern | 17.0 ±3.6 | 5.5 ±2.1 | 12.7 ±0.8 |
| 40 % intern | 19.2 ±3.4 | 14.5 ±6.3 | 17.0 ±3.6 |
| 60 % intern | 32.3 ±5.5 | 16.5 ±1.6 | 18.5 ±1.2 |
| 80 % intern | 36.7 ±2.6 | 25.5 ±9.8 | 16.0 ±1.5 |
| 100 % intern | 40.0 ±3.2 | 101.7 ±9.8 | 17.5 ±1.2 |

CCS: croscarmellose sodium, CPV: crospovidone, Mgst: magnesium stearate, PCM: paracetamol, PEG: polyethylene glycole, SDO: colloidal silicium dioxide, SSG: sodium starch glycolate
